# Letter from Elgin

**Published:** 1883-02

**Authors:** H. Rosencrans

**Affiliations:** Elgin


ARTICLE VI.

ELGIN, JAN. 30, 1883.

S. M., age about fifty-five years, while spading in his garden in this place, the ground being dry and hard, ruptured himself very severely. This occurred on the 20th of last May. It appears that it was an old hernia, right inguinal, as it had appeared once before, but not so bad, and he had been wearing a sort of home-made truss. The method adopted, as I was informed, to reduce it before, had been to stand the patient on his head, or nearly so, holding him in that position by two men, while the surgeon of St. C. manipulated to reduce the hernia. This method of procedure doubtless impressed those present as being a very scientific way of doing it. The method adopted by me was to have the hips of the patient well elevated in the bed; I administered to him a brandy toddy, applied an ice-bag over the extent of the tumor, leaving this on forty minutes; by this time all pain had disappeared. I then, with both hands extended over the tumor, commenced a firm and steady pressure in the direction of the inguinal ring, reducing the hernia in about thirty minutes. This hernia was immense, extending down between his thighs, obliterating penis and scrotum, or involving them in the swelling so as to leave no natural appearance. Dr. B., homœopathist, was present with me, being a brother-in-law of the patient.

H. ROSENCRANS, M.D.

